# Integrative comparative proteomics identifies core differentially expressed proteins and pathways for drought tolerance by exploiting contrasting rice varieties

**DOI:** 10.3389/fpls.2026.1742602

**Published:** 2026-03-06

**Authors:** Qiong Kuang, Shiyan Huang, Shiquan Bian, Jinggui Jiang, Mingyuan Chen, Junhua Liu, Shufan Lei, Zhiwen Lv, Liping Gan, Chengzhi Huang

**Affiliations:** 1 College of Biological and Food Engineering, Chongqing Three Gorges University, Wanzhou, China; 2 Rice Research Department, Chongqing Three Gorges Academy of Agricultural Sciences, Wanzhou, China; 3 Rice Research Institute, Anhui Academy of Agricultural Sciences, Hefei, China

**Keywords:** antioxidant enzymes, drought stress, metabolic pathways, PPI network, proteomics, rice

## Abstract

Drought stress has been a severe challenge to world rice production. To elucidate the underlying mechanisms for rice drought adaptation, physiological characteristics, transcript expression patterns of related genes, and protein profiles from rice leaves were established for four rice cultivars with drastically different degrees of drought tolerance, and under drought stress, tolerant cultivars presented significantly reduced damage in growth and biomass retention. The loss in their main physiological characteristics was less than half of that in the sensitive varieties. Physiologically, the two tolerant varieties (303B and HY796) exhibited 30%–50% higher antioxidant enzyme activities, such as catalase (CAT), peroxidase (POD), and superoxide dismutase (SOD), and a lower malondialdehyde (MDA) level, indicating effective cell membrane protection. Furthermore, there were also more strongly upregulated drought-responsive genes (such as *OsDSM1*, *OsCPK9*, and *OsSNAC1*) between the two varieties. A more tightly coordinated response was revealed at the protein level in the tolerant varieties via proteomic analysis. Carbohydrate metabolism, redox homeostasis, and protein transport were found to be activated in the tolerant varieties via functional enrichment analysis, while photosynthesis was suppressed. However, the networks of protein–protein interaction between more drought-tolerant lines were richer and well-connected. In the interaction networks of proteins, these two drought-tolerant lines had key hub proteins that play pivotal roles in effective signal transduction. The results systematically presented the complex regulatory network basis of drought resistance in rice and offered novel molecular targets for screening drought tolerance in rice.

## 1 Introduction

Rice (*Oryza sativa* L.) is the most important crop in the world, a staple for over half of the world population (Lou et al., 2025), but its productivity is severely threatened by abiotic stresses: drought is currently the most severe abiotic limiting factor, particularly in rain-fed crop systems (Feng et al., 2023). In light of increasing impacts of climate change that trigger more severe and frequent droughts, breeding drought-tolerant rice varieties becomes imperative if the yields are to be sustained and the world’s food security is to be ensured (Khan et al., 2021). With drought continuing to be a recurring and increasing threat to global rice production, and breeders desperately needing to produce drought-tolerant varieties, we argue for increased integrated knowledge to unravel the molecular basis of drought tolerance.

Drought stress can hinder various physiological and biochemical processes in rice, such as photosynthesis, nutrient acquisition, and growth, and finally causes severe yield loss (Yu et al., 2024). A variety of genes and regulatory networks are involved in the molecular mechanisms that allow rice to respond to drought (Han et al., 2024; Ye et al., 2025). For example, key drought-responsive genes include NAC transcription factors (e.g., *OsNAC1*), which regulate stress signaling pathways; calcium-dependent protein kinases (e.g., *OsCPK9*), which participate in phosphorylation cascades; and ethylene-responsive transcription factors (e.g., *OsERF71*), which modulate adaptive responses to drought stress (Lee et al., 2017; Qu et al., 2022; Shi et al., 2025).

Transcriptomics studies have already provided insights into changes in gene expression during stress conditions (Pal et al., 2022; Kaur et al., 2023). Although genes play a critical role in modulating responses at the transcriptomic level, and quantitative trait locus (QTL) explains the heritability to measure trait at a genomic level (Su et al., 2015; Tang et al., 2018), the proteome, the functional executor that mediates physiological responses of adaptation, reveals the molecular view in a simple way to understand the mechanism of how rice develops during drought (Singh and Jwa, 2013; Gujjar et al., 2025). At the protein level, drought stress commonly induces the upregulation of antioxidant enzymes—such as catalase and peroxidase—to scavenge reactive oxygen species (ROS), as well as molecular chaperones that help maintain protein homeostasis, both of which are essential for cellular protection (Gorripati et al., 2021; Raza et al., 2025). Since proteins sense stress, produce signals, and build adaptive mechanisms, proteomic analysis plays an important role in unraveling these molecular networks (Li et al., 2018). Moreover, protein–protein interaction (PPI) networks have emerged as a powerful approach for identifying hub proteins and functional modules that orchestrate stress responses, including those governing carbohydrate metabolism and redox homeostasis (Merumba et al., 2025). Recently, the development of proteomic tools centered on high-quality mass spectrometry has given us the ability to completely resolve the time variation of stress response proteins (Mansour and Hassan, 2022; Mesgarzadeh et al., 2022; Susanto et al., 2025).

A multitude of studies have demonstrated that the protein expression of multiple proteins associated with energy metabolism, ROS scavenging, and protein turnover is drastically altered in response to drought (Mahmud et al., 2023; Rahman et al., 2024), e.g., the downregulation of PSII and ATP synthase complexes to keep photosynthesis in check, the upregulation of chaperones, and antioxidant enzymes to protect photosynthesis from oxidative stress (Valero-Galvan et al., 2013; Dahal et al., 2014). Notably, most studies have been conducted on a particular genotype or within limited genetic backgrounds, focusing on phenotypic traits between a single genotype and a particular range of the background under severe treatment that is incompatible with a real situation in the field (Wei et al., 2024). Meanwhile, post-translational modification (e.g., phosphorylation) has been considered a vital regulator in drought stress pathways (Soma et al., 2021; Zhu et al., 2024). Comparing genotypes with vast genotype-to-phenotype (GTPh) data is increasingly believed by more rice researchers to unveil the special drought-adaptive mechanisms that provide rice with tolerance (Hickey et al., 2022; Ghorbanzadeh et al., 2023; Sorwar Jahan et al., 2025). For example, increased protein expression in osmotic adjustment, redox balance, and cell wall strengthening molecules was found in drought-tolerant compared with drought-sensitive varieties, and by contrast, photosynthesis-related proteins were much lower in drought-sensitive varieties (Nounjan et al., 2018; Bhardwaj et al., 2021; Jin et al., 2024; Zhao et al., 2025). Our study also provides an integrated comparative proteomic approach aimed at clarifying these mechanisms.

In this work, we performed a parallel proteomic analysis of four rice cultivars under drought and control treatments. Our prediction was that drought-tolerant cultivars would trigger a more active adaptive response, which would be reflected in terms of higher antioxidant enzyme activity, in certain selective upregulation of proteins involved in osmoprotection and stress signaling, and in the recalibration of important pathways such as carbohydrate metabolism and phenylpropanoid biosynthesis. Therefore, using the high-throughput label-free Liquid Chromatography-Tandem Mass Spectrometry (LC–MS/MS) method, we attempted to identify the genotype-specific proteomic patterns and the core regulatory proteins, and combining the network of protein–protein interactions with physiological data and key drought-responsive gene expression levels, we attempted to deduce the coordinated mechanisms of drought tolerance. We present the multidimensional molecular resource and the candidate targets for breeding stress-tolerant rice varieties.

## 2 Materials and methods

### 2.1 Plant material and growth conditions

Four rice (*O. sativa* L.) germplasms (303B, HY796, WH99, and 302B) with different degrees of drought tolerance were studied. These cultivars were selected based on performance under drought stress at the Drought Phenotyping Platform of the Chongqing Three Gorges Academy of Agricultural Sciences. Specifically, 303B and HY796 are highly drought-resistant, maintaining stable grain yields under moderate drought, whereas 302B and WH99 are drought-sensitive, exhibiting significant yield reductions under the same stress regime. All four lines belong to the indica subspecies but differ in breeding origin: 303B and 302B are indica-type maintainer lines, and WH99 is an indica hybrid restorer line; all three were developed by the Chongqing Three Gorges Academy of Agricultural Sciences. HY796 is a conventional indica variety originally bred by Shanghai Tiangu Biotechnology Co., Ltd. This combination of contrasting drought phenotypes and genetic diversity provides a robust foundation for comparative proteomics aimed at identifying core drought-responsive proteins.

A pot experiment was carried out in a rainout shelter at the experimental station of the Chongqing Three Gorges Academy of Agricultural Sciences (30.81°N, 108.41°E) in the rice growth season of 2024. Plants were grown in plastic pots (height 25 cm, diameter 30 cm) with 10 kg of paddy soil–sand mixture (3:1 v/v). A randomized complete block design (RCBD) with three replicates was used. The drought stress was applied at the four-leaf stage by restricting the irrigation after maintaining soil moisture at 40% of field capacity, as mentioned by Yang et al. (2022), and while maintaining 100% field capacity for well-watered control.

### 2.2 Determination of biomass and physiological indicators

The biomass and physiological indicators of these rice plants were determined using previously mentioned methods (Qu et al., 2022). Three uniformly growing seedlings were selected from each germplasm and treatment group to determine phenotypic traits, including plant height, root length, fresh root weight, fresh weight of stems and leaves, and root number. The activities of superoxide dismutase (SOD), peroxidase (POD), and catalase (CAT) and the content of malondialdehyde (MDA) in rice leaves were measured using commercially available test kits (Solarbio, Beijing, China). The procedures were slightly modified while following the manufacturer’s protocol.

### 2.3 qRT-PCR analysis

The total RNA was extracted from the rice leaf samples using the method of Trelief^®^ Hi-Pure Plant RNA Extraction Kit (Sangon, Shanghai, China) according to the instructions in order to obtain high-quality RNAs without genomic DNA contamination. The first-strand cDNA was synthesized from the extracted RNA using the method of SynScript^®^ III RT SuperMix (Sangon, Shanghai, China), which can be used as a good template for further quantitative real-time PCR. The expression of genes was analyzed via qRT-PCR using the SYBR Green Real-time PCR Master Mix Kit (Sangon, Shanghai, China) on an ABI QuantStudio 7 Pro system (Thermo Fisher Scientific, Waltham, MA, USA). Relative expression level was calculated using the 2^−ΔΔ^*^Ct^* algorithm according to previous research (Bian et al., 2022). Primers were created with the help of NCBI online tools (https://www.ncbi.nlm.nih.gov/tools/primer-blast/). qRT-PCR primer information is shown in **Supplementary Table S1**.

### 2.4 Protein extraction

Proteins were isolated via phenol extraction based on a previously published method, with some variations (Isaacson et al., 2006). Freshly frozen rice leaf materials were ground under liquid nitrogen until they became a fine powder. Approximately 100 mg of the resulting powder was transferred to a pre-cooled 2-mL centrifuge tube. Briefly, 800 μL of phenol extraction buffer (pH 7.8), supplemented with phosphatase inhibitors and 1 mM phenylmethylsulfonyl fluoride (PMSF), was added to the powder, followed by an equal volume of Tris-saturated phenol (pH 7.8). The mixture was vigorously vortexed before being incubated for 40 min at 4°C with constant shaking. After being centrifuged at 7,100 × *g* for 10 min at 4°C, the upper phenolic phase was recovered carefully. Five volumes of ice-cold 0.1 M ammonium acetate in methanol was added to induce protein precipitation, and the mixture was incubated overnight at −40°C. After centrifugation at 4°C at a speed of 12,000 × *g* for 10 min, the precipitate was collected. Then, the precipitate was rinsed twice with pre-cooled methanol and twice with cold acetone to remove residual methanol and salts. The final protein precipitate was dried at room temperature for approximately 5 min and then dissolved in an appropriate protein lysis buffer. After a brief centrifugation for 10 min (at a speed of 12,000 × *g*), the supernatant was collected as the total protein extract. The sample solution was kept at −80°C until use. Protein concentration was determined using the Bradford assay (Kielkopf et al., 2020) with bovine serum albumin as a standard.

### 2.5 Proteolysis and peptide desalting

The rice leaf protein extraction was carried out according to the procedure described in a previous study (Franz and Li, 2020). The rice leaf protein was treated by protein hydrolysis and peptide desalting; 5 mmol/L DTT was added to the protein solution. The solution was mixed and incubated at 55°C for 30 min. The mixture was cooled to room temperature on ice, and an appropriate volume of iodoacetamide was added to the 10 mmol/L mixture. The mixture was well vortexed and kept in the dark at room temperature for 15 min. Subsequently, six times of volumes of cooled acetone was added and precipitated in the refrigerator (−20°C) overnight. Then, it was centrifuged at 8,000 × *g* for 10 min at 4°C for precipitation. After the remaining acetone was removed via evaporation for 2–3 min, the precipitate was dissolved in 100 μL of 50 mmol/L ammonium bicarbonate solution. Trypsin-TPCK (1 mg/mL) was diluted (1:50 w/w) at 37°C via overnight digestion; the digested sample was freeze-dried and kept at −80°C for further experiment.

The re-collected peptides were deprotonated with the use of the SOLA™ SPE 96-well plate. The plate was first activated twice with 200 µL of methanol at a vacuum flow rate. The equilibration was performed twice with 200 µL of water with 0.1% formic acid at a vacuum flow rate. The sample (50–500 µL) was loaded on the plate at the same vacuum flow rate for deprotonation. The loading process was repeated to further enhance the binding efficiency. Afterward, the plate was further washed twice with 200 μL water solution (0.1% formic acid) to flush away all the impurities; 50% acetonitrile–water with 0.1% formic acid (150 μL) was added, and the peptides were eluted three times to give a final eluate of 450 μL. Finally, the eluate was dried under vacuum.

### 2.6 DIA-based mass spectrometric analysis

Prior to mass spectrometric injection, each sample was spiked with iRT peptides at a volume ratio of 1:20 to serve as an internal standard. An equal amount of peptides from all digested samples was taken, and a suitable aliquot from each sample was subjected to chromatographic separation using a Vanquish Neo UHPLC system (Thermo Fisher Scientific Inc., Waltham, MA, USA) as described before (Sansoucy et al., 2021). Mobile phase A comprised 0.1% formic acid dissolved in water, and mobile phase B was 0.1% formic acid in 80% acetonitrile–water. The gradient elution program was set as follows: at 0 min, 4% B; at 0.2 min, 25% B; at 4 min, 75% B; at 5.8 min, 65% B; and at 6.9 min, 99% B. After separating the peptides, data-independent acquisition (DIA) mass spectrometric analysis was carried out using an Orbitrap Astral mass spectrometer (Thermo Fisher Scientific Inc., Waltham, MA, USA). The mass spectrometry parameters were adjusted to the following: the resolution of the Orbitrap was set to 240,000, full MS scan range spanned 380–980 m/z, Automatic Gain Control (AGC) target was 5e3, maximum injection time was 5 ms, MS/MS scan range covered 150–2,000 m/z, MS/MS AGC target was 4e3, MS/MS maximum injection time was 3 ms, RF lens was set to 0.4, isolation window was 2 m/z, Higher-Energy Collisional Dissociation (HCD) collision energy was set to 0.26, and cycle time was 0.6 seconds.

### 2.7 Proteomic analysis

The proteomic analysis was conducted according to the methods described in previous studies (Tappiban et al., 2021; Khan et al., 2024). All mass spectrometry data were merged using the software DIA-NN to complete the database retrieval of DIA mass spectrometry data and the quantitative analysis of protein DIA. The database search sequence file was uniprotkb_proteome_*O. sativa* subsp.*indica*-39946_2024_11_06.fasta. The analysis parameters of the DIA-NN software were set as follows: the enzyme was trypsin, with a maximum allowable number of missed cutting sites of 1. Its fixed modification was carbamidomethyl (C), and its variable modifications were methionine oxidation (Oxidation, M) and protein N-terminal acetylation (acetyl, protein N-term). The database mode was Target-Reverse, and the false discovery rate (FDR) of both peptide-spectrum matching (PSM) and protein FDR was set to 0.01. Reliable protein analysis and data quality control were performed. The original data were retrieved from the database. Unique peptides were ≥1, the number of valid values of all samples were ≥2, at least one group of proteins with a valid value proportion ≥50% was retained, the groups with the mean of the same group for the effective value proportion ≥50% were filled, and the remaining blank values were filled with half of the minimum value of the matrix. Reliable proteins were obtained via median standardization and log_2_ logarithmic transformation.

The differentially expressed proteins (DEPs) were identified using *p*-value <0.05 and absolute fold change (FC) >2. Namely, those proteins satisfying a *p* < 0.05 and an FC > 2 were considered upregulated, while those proteins satisfying a *p* < 0.05 and an FC < 1/2 were considered downregulated. Gene Ontology (GO) was used to classify DEPs according to three functional categories, namely, biological process (BP), cellular component (CC), and molecular function (MF). Kyoto Encyclopedia of Genes and Genomes (KEGG) pathway enrichment analysis was employed to discover the key pathways of DEPs. Moreover, the PPI network of DEPs was built using the STRING database, and the interaction networks were analyzed to identify the potential functional modules and hub proteins.

### 2.8 Statistical methods

Data processing, analysis, and visualization were performed using GraphPad Prism 8.0 (GraphPad Software, Boston, MA, USA), which was also employed for statistical data analysis. For statistical tests and data plotting, SPSS 27.0 (IBM Corp., New York, NY, USA) was used. Student’s *t*-test was used to find differences of means, which were considered significant if *p < 0.05 and **p < 0.01.

## 3 Results

### 3.1 Phenotypic and biomass analyses of rice varieties

**Figure 1:**
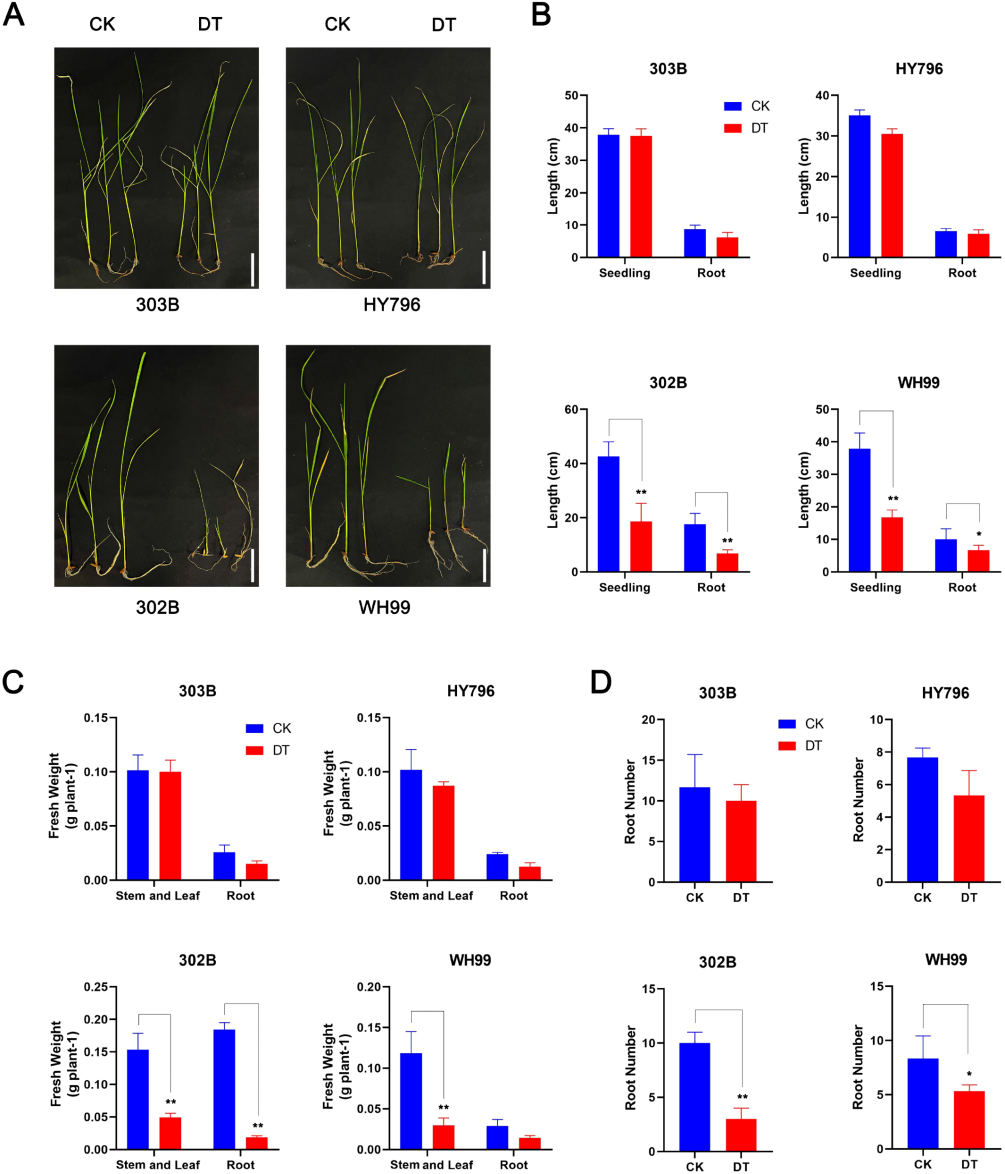
Phenotypic and biomass analyses of rice varieties. **(A)** Phenotypic photographs of four rice varieties (303B, HY796, 302B, and WH99) under drought treatment (DT) and control (CK) conditions. Scale bars, 5 cm. **(B)** Statistical analysis of seedling and root lengths of the four rice varieties under DT and CK conditions. **(C)** Statistical analysis of fresh weight (FW) of stems, leaves, and roots of the four rice varieties under DT and CK conditions. **(D)** Statistical analysis of root number of the four rice varieties under DT and CK conditions. Error bars indicate standard deviations, and asterisks denote significant differences between CK and DT (*p < 0.05, **p < 0.01) as determined using Student’s *t*-test.

To evaluate the drought-resistant phenotypes, four rice lines (303B, HY796, 302B, and WH99) were grown under drought treatment (DT) and control (CK) treatment, and their growth traits were systematically studied. As shown in **Figure 1A**, significant changes in phenotypes occurred among the four rice varieties under drought stress, while the two drought-tolerant varieties of rice, i.e., 303B and HY796, exhibited virtually normal growth in terms of morphology with mild reductions in the growth-related traits.

Further quantitative analyses confirmed the clear performance gap between drought-tolerant and drought-sensitive rice, as under DT, both tolerant 303B and HY796 remained highly stable in growth parameters; no statistically significant difference for seedling length (**Figure 1B**) was detected in CK, and there was minimal loss for root number (**Figure 1D**). On the contrary, highly drought-sensitive 302B presented higher vulnerability, that is, severe, highly significant reductions in all indices, such as 42.3% in seedling length, 38.7% in root length, 52.1% in stem–leaf fresh weight, and 47.6% in root number, compared with CK.

The intermediate type WH99 displayed a contradictory response pattern: on the one hand, the decrease of seedling length was extremely significant, and on the other hand, the root length decreased significantly, which suggests some adaptation mechanism to manage the stress. Especially striking was the capacity to retain fresh weight: stem–leaf biomass of the tolerant 303B and HY796 retained more than 85% of their original biomass under DT, indicating a remarkable capacity for water retention and osmotic adjustment. In contrast, the sensitive 302B and WH99 retained only approximately 51% and 70% of their biomass, respectively, reflecting severe water loss and tissue dehydration (**Figure 1C**).

All these comparative analyses clearly indicate that drought-tolerant genotypes focus on the resource allocation toward root maintenance for conserving the above-ground biomass and structural integrity of the root system, as opposed to drought-sensitive genotypes, which undergo complete collapse of plant growth. Clearly, these comparative analyses indicate that drought-resistant lines employ allocation for maintaining aboveground and the structural survival of roots against the complete breakdown in growth in the sensitive ones. This hierarchical tolerance ranking produced from these phenotypic assays (which classified 303B and HY796 as tolerant, WH99 as mildly tolerant, and 302B as susceptible) will be pivotal to future studies on molecular mechanisms underlying these disparate adaptive approaches.

### 3.2 Physiological and transcriptional responses of rice varieties to drought stress

**Figure 2:**
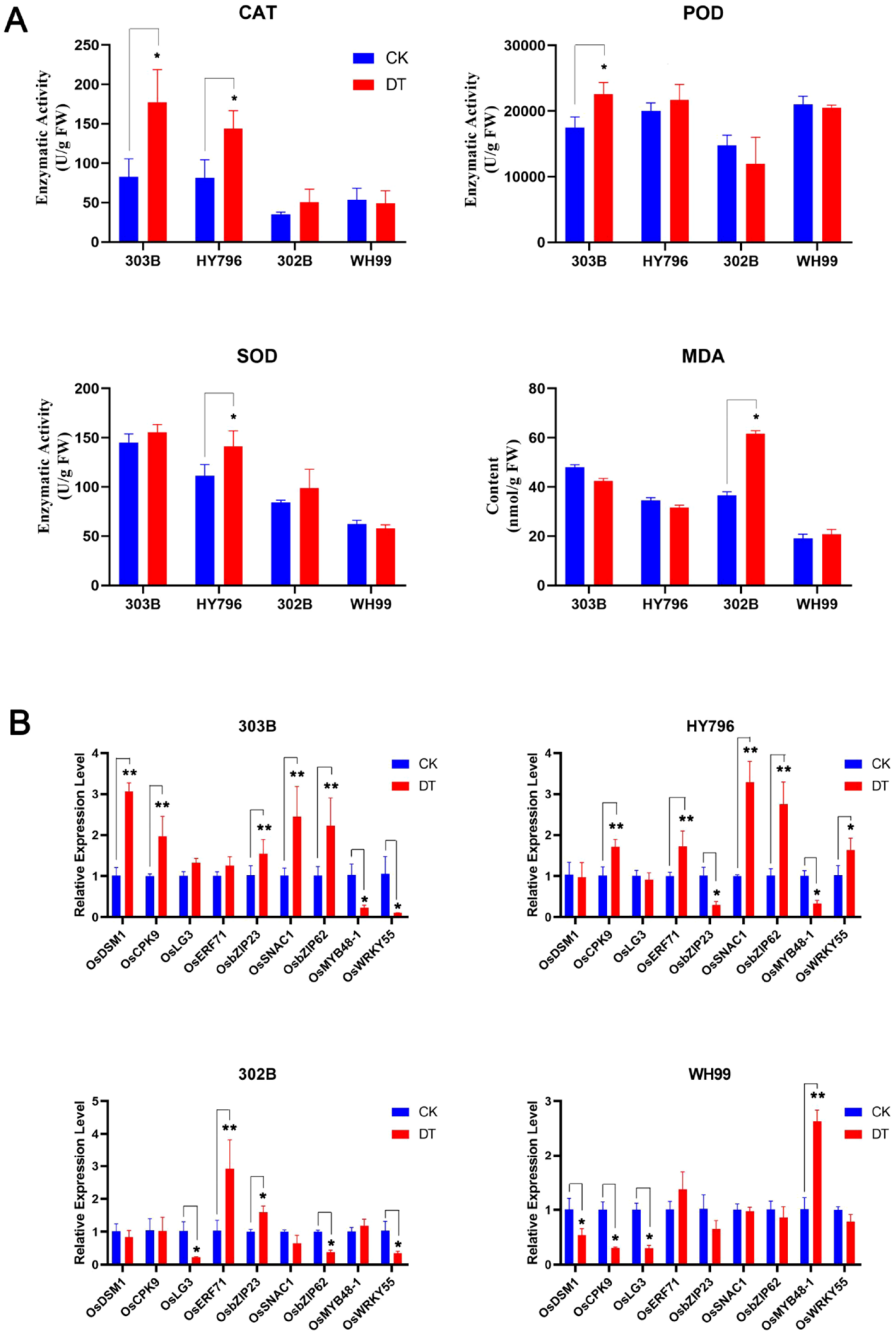
Physiological and transcriptional responses of rice varieties to drought stress. **(A)** Activities of catalase (CAT), peroxidase (POD), superoxide dismutase (SOD), and malondialdehyde (MDA) content in rice varieties 303B, HY796, 302B, and WH99 under control (CK; blue bars) and drought treatment (DT; red bars). **(B)** Relative expression levels of key stress-responsive genes in the four rice varieties under CK and DT. In both panels (**A, B**), error bars indicate standard deviation, and asterisks denote significant differences between CK and DT (*p < 0.05, **p < 0.01) as determined using Student’s *t*-test.

We further investigated the responses of those rice varieties to drought stress at the physiological level by measuring the activities of three primary antioxidant enzymes (CAT, POD, and SOD) and MDA contents, as well as the stress-related gene expression pattern under DT and CK conditions among four rice varieties, as shown in **Figure 2**.

Tolerant lines 303B and HY796 showed strong, consistent responses for physiological and transcriptional level changes upon drought stress, as compared to the susceptible lines, which correlated to their drought tolerance shown in **Figure 1A**. Both lines 303B and HY796 have significantly increased activity levels of CAT, POD, and SOD under DT (**Figure 2A**), thus strengthening the antioxidant system, enabling ROS scavenging, and maintaining low levels of MDA, which indicate minimal membrane damage from oxidative stress under water deficit and corroborate the superior cellular water status observed in the biomass data (Ren et al., 2025) (**Figure 1C**).

To bridge the physiological observations with transcriptional regulation, we selected four key drought-responsive genes (*OsDSM1*, *OsCPK9*, *OsSNAC1*, and *OsERF71*) for qRT-PCR analysis (**Figure 2B**). We chose these genes as they are among the most frequently reported master regulators of drought tolerance in rice, and their known functions in stress signaling (*OsCPK9* and *OsERF71*), osmotic adjustment (*OsDSM1*), and transcriptional control (*OsSNAC1*) are highly relevant to the proteomic and phenotypic patterns that we aimed to decipher (Lee et al., 2017; Qu et al., 2022; Ahmad et al., 2022; Shi et al., 2025).

Gene expression analysis suggested that coordinated physiological resilience in tolerant lines was characterized by concomitant induction of major drought-responsive genes. For these lines, *OsDSM1* and *OsSNAC1* were induced at 3.0–3.2-fold and 2.5–3.0-fold, respectively, and *OsCPK9* and *OsERF71* were also induced substantially (**Figure 2B**). In WH99, which is mildly tolerant, there was upregulation of antioxidant enzymes but not fully: a modest upregulation of MDA and a selective upregulation of genes consistent with the moderate tolerance. In contrast, the sensitive variety 302B displayed complete failure of defensive mechanisms, such as little upregulation of enzymes; 2.8-fold increase in MDA, which signals a breakdown of the membrane; and disturbance of the expression of relevant genes (**Figure 2B**). The relationship was evident among all cultivars: the consistency between physiological and transcriptome response was proportional to the gradient of drought tolerance. The presented results provide a molecular basis for the phenotypic distinction and pave the way for further proteomic search into the central proteins driving drought adaptation.

### 3.3 Identification of differentially expressed proteins

We performed LC–MS/MS-based proteomic analysis of the four rice varieties grown under both drought and control conditions. Database searches of the resulting spectra identified 9,432 proteins and 92,594 peptides, generating a comprehensive dataset for subsequent differential analysis (**Supplementary Table S2**).

**Figure 3:**
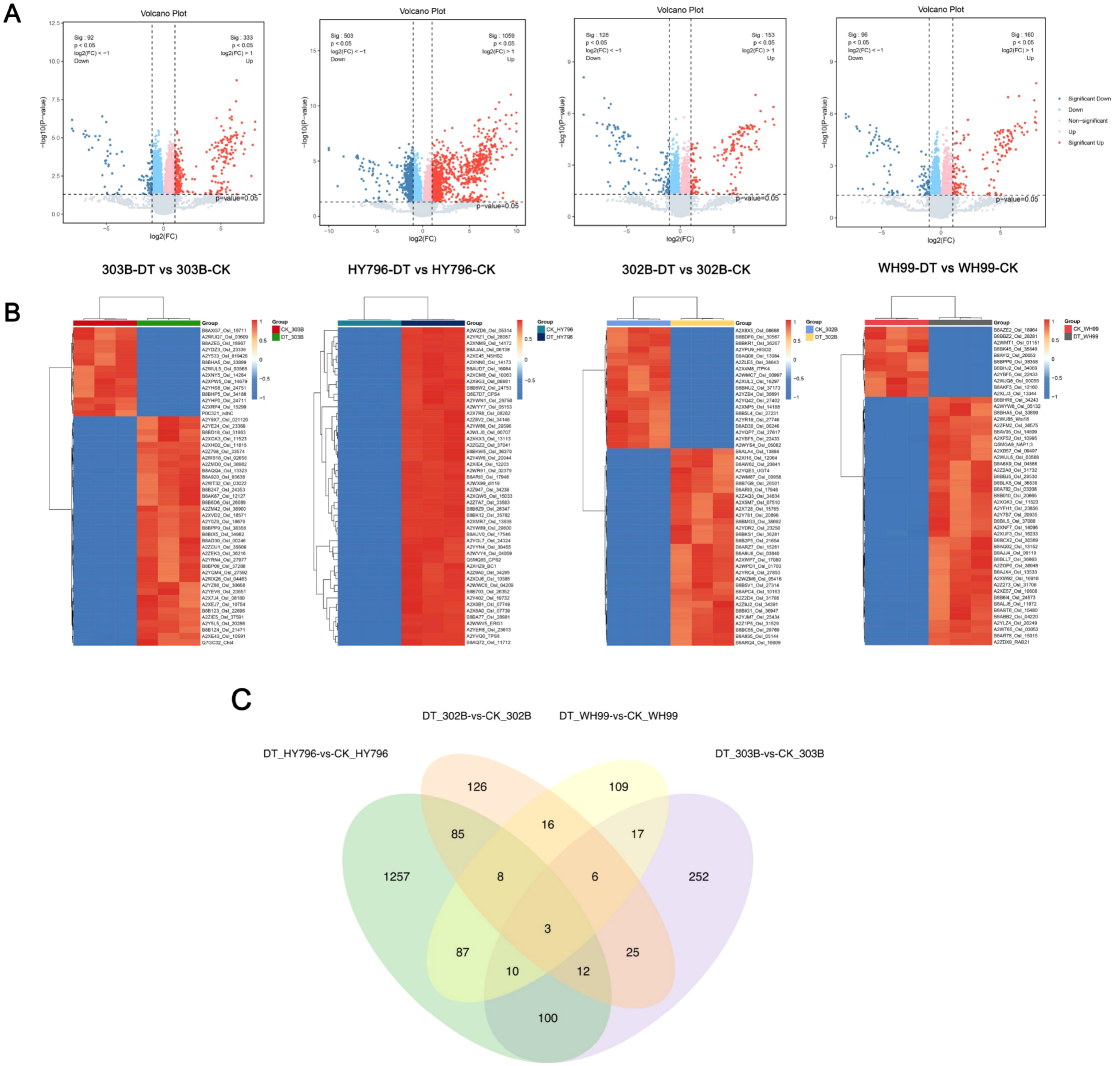
Proteomic analysis of differentially expressed proteins (DEPs) in four rice varieties under drought treatment (DT) and control (CK) conditions. **(A)** Volcano plots depicting protein expression differences between DT and CK groups for 303B, HY796, 302B, and WH99. The x-axis represents log_2_(fold change, FC), and the y-axis represents –log_10_(*p*-value). Red dots indicate significantly upregulated DEPs (|log_2_FC| > 1, *p* < 0.05), blue dots indicate significantly downregulated DEPs, and gray dots indicate non-significant proteins. **(B)** Heatmaps showing hierarchical clustering of DEPs across DT and CK samples for each variety. Rows represent proteins, columns represent samples, and color gradients (red for high expression and blue for low expression) illustrate relative protein abundance, reflecting variety-specific expression patterns under drought stress. **(C)** Venn diagram illustrating the overlap of DEPs among the four varieties, with different colored regions representing the number of unique or shared DEPs, highlighting genotype-specific and conserved drought-responsive protein sets.

DEPs in the distribution and overlap across the four rice varieties are summarized in **Figure 3**. In each of the varieties, comparison of the DT group and CK group showed large numbers of DEPs (defined as |log_2_(FC)| > 1 and *p* < 0.05), with highly upregulated and downregulated proteins colored red and blue, respectively (**Figure 3A**). The DEPs clustered hierarchically into separate groups for the DT and CK samples in each of the two varieties, indicating genotype-specific responses of changes in protein abundance under drought conditions (**Figure 3B**). In addition, the overlap of DEPs between the four varieties on the Venn diagram indicated that only a few DEPs were in common across all four varieties (**Figure 3C**). HY796 showed the most novel DEPs (1,257), and only 100 DEPs were shared between all varieties. This indicated a clearly genotype-dependent proteomic drought response.

Taken together, these results show the specific and genotype-dependent modifications caused by drought at the proteome level. This study, therefore, lays a preliminary basis for future investigations on identifying the most central functional proteins and the corresponding regulatory network that determines drought tolerance.

### 3.4 Gene Ontology enrichment analysis

**Figure 4:**
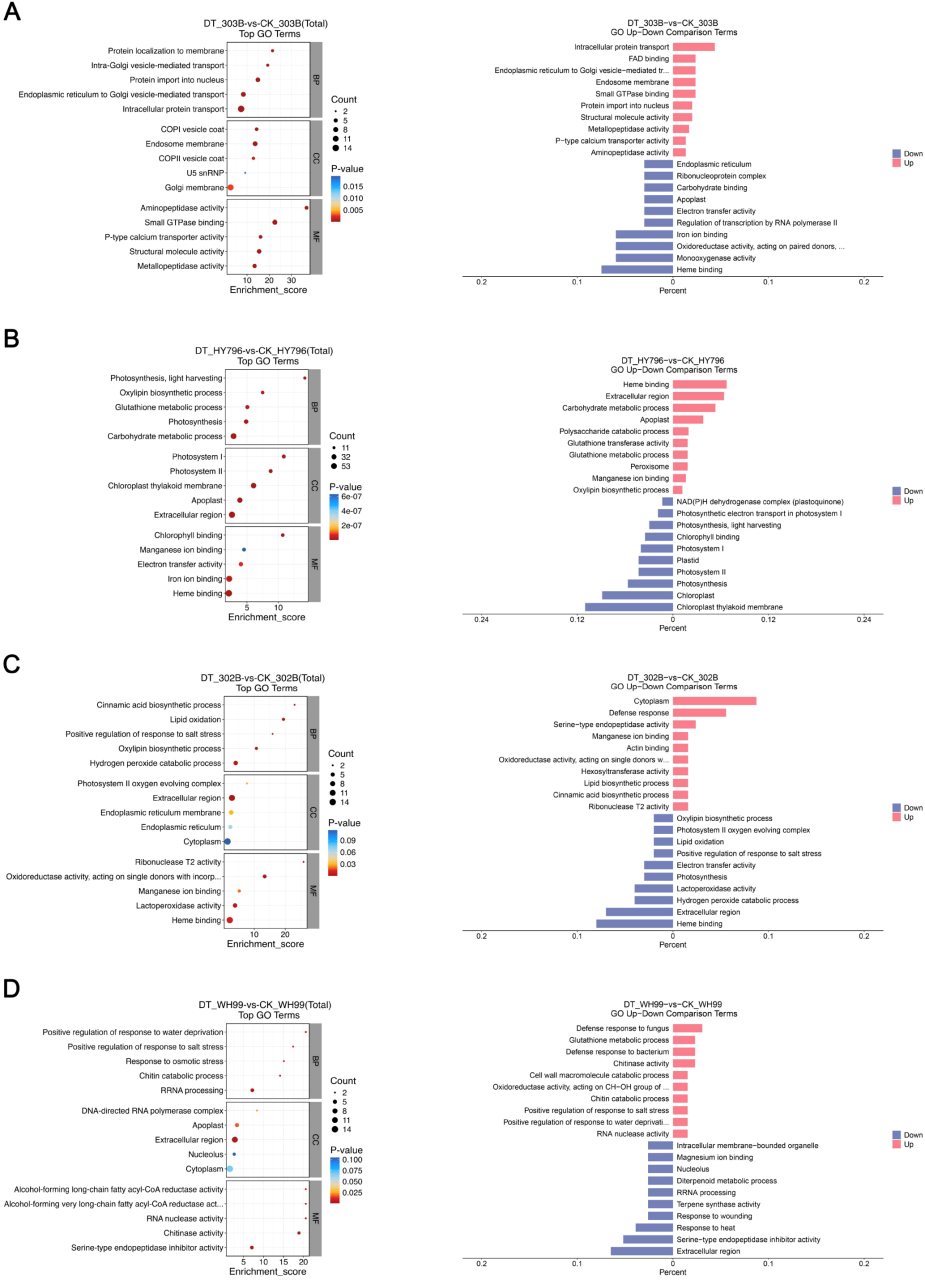
Gene Ontology (GO) enrichment analysis of differentially expressed proteins (DEPs) in four rice varieties under drought stress. **(A–D)** GO enrichment profiles for 303B, HY796, 302B, and WH99, respectively, comparing drought treatment (DT) and control (CK) groups. For each variety, the left panel is a bubble plot illustrating the top 30 enriched GO terms across biological process (BP), cellular component (CC), and molecular function (MF) categories: bubble size corresponds to the number of DEPs annotated to a term, color intensity reflects the statistical significance (*p*-value), and the x-axis represents the enrichment score. The right panel is a bar plot comparing the percentage of upregulated (red) and downregulated (blue) GO terms.

In order to obtain functional interpretations, the drought-responsive DEPs of each cultivar were further subjected to GO enrichment analysis in three GO categories, including the BP, CC, and MF categories (**Figure 4**; **Supplementary Table S3**), and thus revealed the biological function perturbation most influenced by drought stress at the protein level.

The difference in the types of responses to drought between the varieties was also apparent from a GO term enrichment comparison (**Figure 4**): the over- and downregulated DEPs in 303B were enriched in functions related to protein intracellular transport and signaling, as well as gene expression, respectively. However, HY796 showed a strong induction of the metabolic detoxification through glutathione metabolism, as well as a strong downregulation of the photostatic apparatus elements. In the sensitive cultivar 302B, the most overinduced metabolism corresponded to defenses and lipid metabolism, while the most downregulated was again photosynthesis. The moderately tolerant WH99 clearly showed a different profile than the rest, with functional categories being regulated for the water-withholding response, and the regulation of oxidoreductase activity ranking higher than diterpenoid metabolism.

These findings collectively indicate that the metabolically regulated pathways (functional capabilities) in a rice genome are restructured by drought stress by changing primarily pathways of stress response regulation, photosynthesis, and intercellular communication, while regulating metabolic status through heme-binding proteins. Such GO-based functional profiling thus provides insights into the key biological processes controlling the drought adaptability in rice.

### 3.5 Kyoto Encyclopedia of Genes and Genomes metabolic pathway analysis

**Figure 5:**
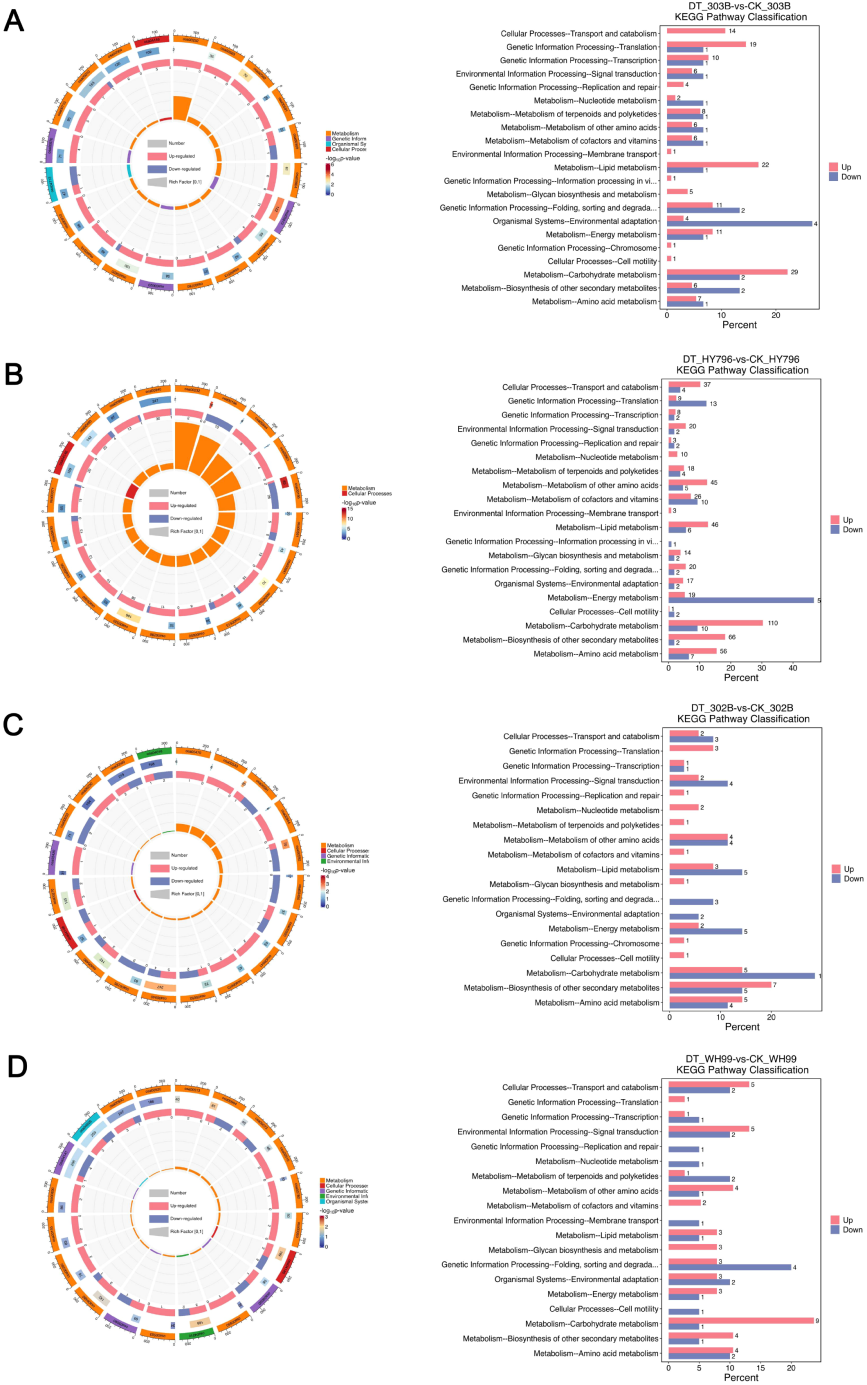
Kyoto Encyclopedia of Genes and Genomes (KEGG) enrichment analysis of differentially expressed proteins (DEPs) in four rice varieties under drought stress. **(A–D)** KEGG enrichment profiles for 303B, HY796, 302B, and WH99, respectively, comparing drought treatment (DT) and control (CK) groups. For each variety, the left panel is a bubble plot illustrating the top KEGG terms: bubble size corresponds to the number of DEPs annotated to a term, color intensity reflects the statistical significance (*p*-value), and the x-axis represents the enrichment score. The right panel is a bar plot showing the KEGG pathway classification, with red and blue bars indicating the percentage of upregulated and downregulated DEPs in each pathway category, respectively.

The level 2 pathways in which the DEPs were involved were found systematically via KEGG enrichment analysis (**Figure 5**; **Supplementary Table S4**). As shown in **Figure 5**, we recharged the level 2 pathway that the DEPs enriched and gave the details of enrichment (enrichment score, number of proteins, and *p*-value) for the DEPs’ regulation ratios of the key pathways.

KEGG enrichment analysis indicated an interplay of conserved and variety-specific pathway adaptations to drought. In HY796, a prominent metabolic rewiring pattern, including upregulated carbohydrate metabolism, amino acid metabolism, and biosynthesis of secondary metabolites, may help to osmotically adjust and detoxify. In comparison, the translation and energy metabolism pathways were downregulated, which suggested an economical shift toward stress response (**Figure 5B**). In contrast, cultivar 303B exhibited a markedly different response, with significant upregulation of genes involved in lipid metabolism (**Figure 5A**). Pathways associated with environmental adaptation were tightly and evenly regulated in this variety. By comparison, the moderate responders WH99 and 302B displayed a considerably weaker transcriptional response. The weak changes in metabolized pathways revealed by WH99 presented some enrichment mainly in the *N*-glycan and diterpenoid biosynthesis, with an upregulation more evident in carbohydrate metabolism (**Figure 5D**), just like 302B with upregulation in the exopolysaccharide and the flavonoid biosynthesis in addition to a weak downregulation of lipid and energy metabolism, hinting at a moderate metabolic change on stress (**Figure 5C**).

Together, KEGG pathway analysis uncovered that rice uses both conserved and lineage-specific metabolic responses under drought. A globally upregulated carbohydrate metabolism was conserved and may prove critical for the regulation of energy and carbon homeostasis. Apart from this general response, the four varieties showed radically different responses in the magnitude and types of regulated pathways, especially in the list of downregulated processes, highlighting the range of adaptation.

### 3.6 InterPro enrichment analysis

**Figure 6:**
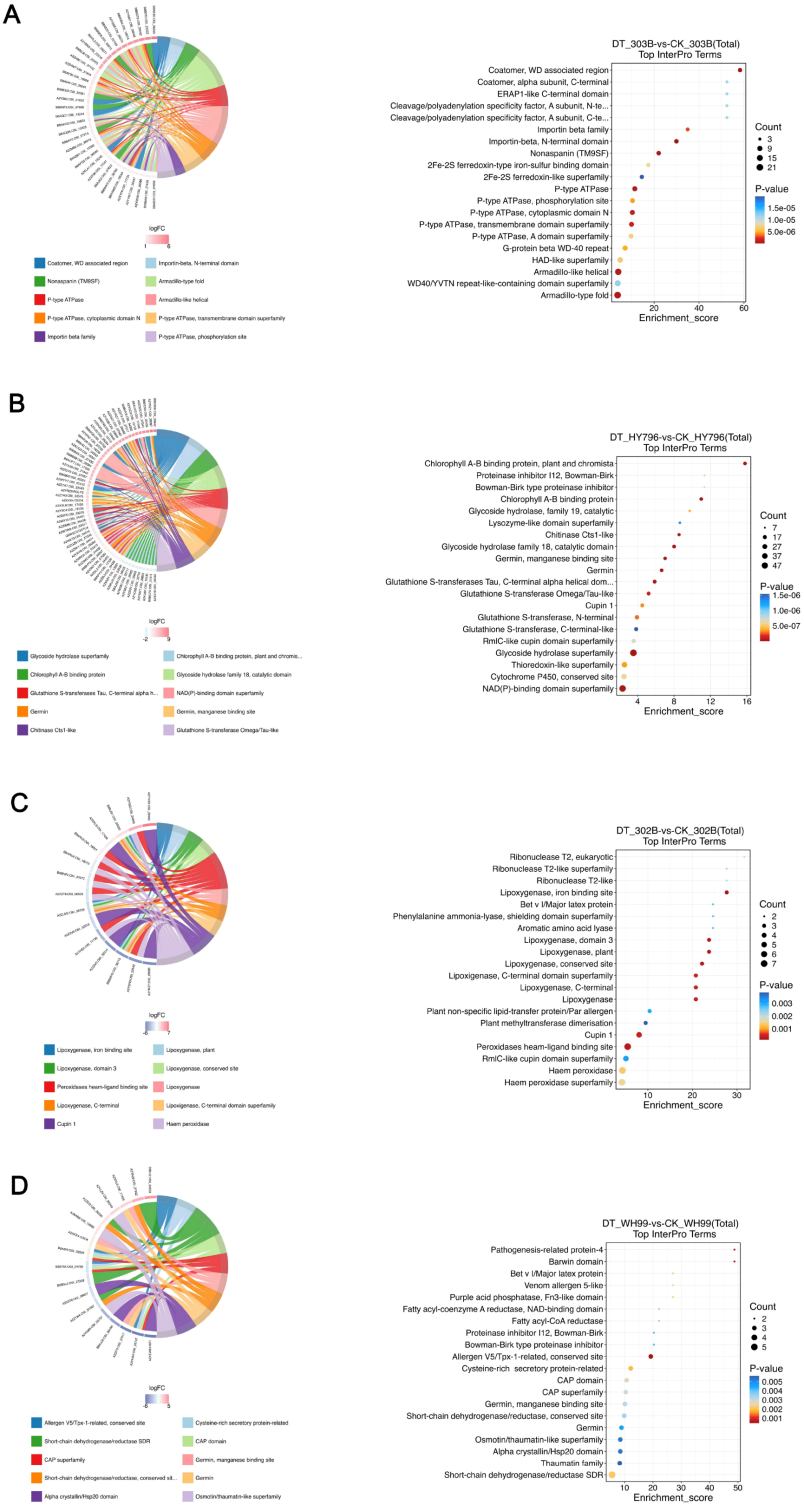
InterPro enrichment analysis of differentially expressed proteins (DEPs) in four rice varieties under drought stress. **(A–D)** InterPro enrichment profiles for 303B, HY796, 302B, and WH99, respectively, comparing drought treatment (DT) and control (CK) groups. For each variety, the left panel is a bubble plot illustrating the top InterPro terms: bubble size corresponds to the number of DEPs annotated to a term, color intensity reflects the statistical significance (*p*-value), and the x-axis represents the enrichment score. The right panel is a bar plot comparing the percentage of upregulated (red) and downregulated (blue) InterPro terms, revealing the distribution of conserved protein domains/motifs in response to drought.

DEPs were also analyzed using InterPro to characterize the families of proteins and the functional domains that they represent, that is, the molecular mechanisms of drought adaptation. Results from the InterPro analysis, which identifies enriched specific InterPro terms for upregulated and downregulated proteins, are presented in **Figure 6** and **Supplementary Table S5**.

The InterPro analysis of 303B indicated a shift in the biological function in the context of drought (**Figure 6A**). Several terms were upregulated that were associated with the Armadillo-type fold domains, which are involved in intracellular transport of stress-regulated proteins and involved in rice growth and abiotic stress tolerance, whereas terms associated with cytochrome P450 (hormone biosynthesis, secondary metabolism, and stress defense) were downregulated. This trend indicates a metabolic shift of use, where the most basic adaptive functions are being “upgraded” with more resources. Based on InterPro, we observed that HY796 responds to drought by upregulating the capacity to hydrolyze carbohydrates and binding redox cofactors in order to provide energy and adjust the metabolism. Conversely, those representing parts of the chlorophyll biosynthetic pathway and light-harvesting complex were downregulated. This is in keeping with a prioritization of resources from photosynthesis to other stress response machinery (**Figure 6B**). The drought-sensitive cultivar 302B also showed the highest level of significantly upregulated InterPro terms, such as lipoxygenase, with a lipoxygenase enzyme participating in lipid metabolism and oxidative stress signaling ^36^, and the shielding domain superfamily of phenylalanine ammonia-lyase, which is involved in the synthesis of barrier lignin and antioxidants ^37^. Terms like eukaryotic ribonuclease T2, which plays a role in RNA degradation,^38^ were downregulated (**Figure 6C**). The inhibition of this RNA metabolic enzyme may divert metabolic resources to the stress response proteins. WH99 upregulated certain categories of protein domains to adapt to drought stress (**Figure 6D**). Differentially upregulated terms were characterized with pathway terms associated with defense against pathogens (pathogenesis-related protein-4) and stress signaling. The most striking phenomenon was the downregulation of small heat shock protein domains, suggesting that WH99 uses alternate pathways for protein preservation. These data also reiterate that drought tolerance is facilitated by unique renetworking at the domain level, which reflects the genotypes’ general physiology and metabolism.

InterPro was used to enrich various response components between accessions. Drought-responsive categories were very diverse: favored protein translocation in 303B toward carbon/nitrogen metabolism (at the cost of photosynthesis) in HY796, lipid/phenylpropanoid pathways in 302B, and defense signaling in WH99. Such discrete domain-level modifications represent functional determinants of drought tolerance and demonstrate the molecular specificity of adaptation in rice.

### 3.7 Differential protein–protein interaction network analysis

**Figure 7:**
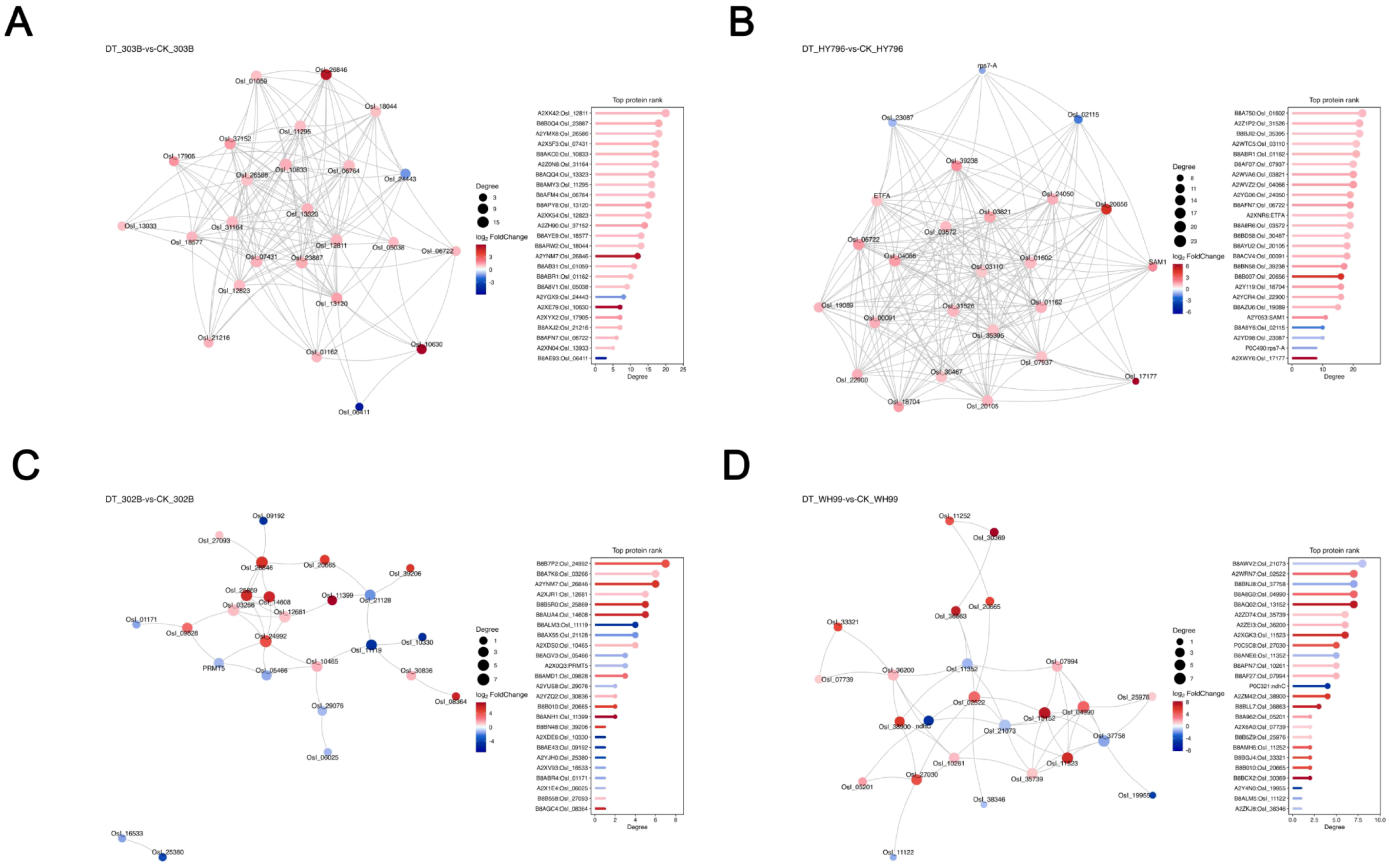
Protein–protein interaction (PPI) networks of differentially expressed proteins (DEPs) in four rice varieties under drought stress. **(A–D)** PPI networks for 303B, HY796, 302B, and WH99, respectively, comparing drought treatment (DT) and control (CK) groups. In each network, node size corresponds to the degree (number of direct protein–protein interactions), and node color represents the log_2_(fold change, FC) of DEPs (red indicates upregulation; blue indicates downregulation). The adjacent panels on the right rank core proteins by degree (highlighting top proteins with the highest connectivity) and display their log_2_FC distribution, emphasizing hub proteins in drought-responsive regulatory networks.

In order to acquire a systems-level insight into drought responsiveness, we constructed PPI networks for the DEPs of each rice variety and compared their network complexity, stability, and functional modularity using the following key topological parameters, including the number of nodes and edges, average node degree, average clustering coefficient, and characteristic path length (**Figure 7**; **Supplementary Table S6**). In these three plots, the nodes were colored by the DEP log_2_ fold change and sized proportionate to the connectivity. These plots make it straightforward to pick out well-connected hub proteins from the image and discern the expression pattern under drought stress.

The PPI network of the tolerant genotypes (303B and HY796) was considerably richer and sturdier than that of the sensitive ones (302B and WH99), with more nodes/edges, a higher mean degree, and a higher clustering coefficient. While in 303B’s network some hubs of high connections were present (i.e., Os1_08946, degree ≥ 9) (**Figure 7A**), in 302B’s network, the interaction between proteins seemed rather sparse (most degree values ≤5) (**Figure 7B**), implying that drought activates a more integrated and modular response in the proteins of a tolerant genotype. The emergent topology with higher modularity and smaller path lengths will give it robustness and rapid signaling ability, leading to fast response to stress.

In the PPI networks of the tolerant forms, there were many prominent proteins by high degree hubs (so-called “top protein rank” panels, **Figures 7A–D**), which play important roles in the center of networks connecting different stress signals and orchestrate the downstream signals in upstream regulatory nodes like the antioxidant defense pathway and carbohydrate metabolic pathway, such as different peroxidases and chaperones in 303B and SAM1 in HY796. This echoes our previous KEGG findings; e.g., the highly linked hub Os1_08946 in 303B is appropriately situated to coordinate ROS scavenging with osmotic adjustment, and SAM1 in HY796 probably regulates polyamine biosynthesis for stress protection. The existence of such hubs endows structural rigidity to the network and leads to a coordinated defense program, which is crucial to the better drought resistance of 303B and HY796 (**Figures 7A, B**).

Overall, our analysis of PPI networks reveals drought tolerance to be better correlated with complex, robust protein networks. The dominant proteins for such networks are the hotpot genes, deserving high research priority for knowledge discovery about the genetic code of drought tolerance and for breeding interventions in drought conditions.

## 4 Discussion

Drought stress causes the maximum constraint on the worldwide rice production; thus, comprehending the mechanisms governing tolerance at various levels and the differences in varietal responses is very important for breeding tolerant cultivars (Liu et al., 2025; Sagar et al., 2025). Here, we report on integrating the analyses of phenotypic, physiological, proteomic, and network-level datasets to ascertain the drought adaptation between the four varieties having different degrees of tolerance. Taken together, our data illustrate the superiority of drought tolerance resulting from a coordinated response in the growth, physiology, proteome, and network robustness, and not from isolated responses by individual molecules. These are results that build upon, while also extending, existing models of cereal drought tolerance and thereby highlight genotype-specific responses that may inspire breeding strategies in the future. Phenotypic and biomass measurements determined a well-defined hierarchy in degrees of tolerance: 303B and HY796 are tolerant, WH99 is intermediate tolerant, and 302B is sensitive (**Figure 1**). The salient characteristic of the tolerant cultivars was the capacity to conserve above-ground biomass and to not destroy root architecture despite the drought, retaining >85% of the stem–leaf fresh weight with respect to >50% in 302B. This reflects a strategic allocation of resources to sustain growth rather than undergo stress-induced collapse, a recognized trait of drought-tolerant plants (Chandregowda et al., 2023; Du et al., 2024). For example, prior work in wheat and maize has also shown that tolerant genotypes prioritize carbon allocation to maintain shoot and root structure (Dreccer et al., 2009; Gao et al., 2023). The WH99 was moderately tolerant with an intermediate phenotype characterized by intermediate decrease of the seedling length but intermediate loss of biomass, pointing to a partial activation of tolerance mechanisms that could have been associated with a growth trade-off.

The physiological and transcriptional results were consistent with the order of tolerance obtained from the genotypes based on the phenotypic values. The two tolerant cultivars, 303B and HY796, showed good coordination and upregulated CAT, POD, SOD, and stress-related genes (*OsDSM1*, *OsSNAC1*, etc.) that supported the efficient ROS scavenging, as well as the membrane stability by maintaining low levels of MDA (**Figure 2**) (Yu et al., 2017; Yin et al., 2024; Tan et al., 2025). Conversely, in the sensitive variety 302B, an aberrant response was observed (low enzymatic activity, increased MDA content, and compromised gene expression), emphasizing the fact that drought resistance requires coordination between different biological levels. The intermediate phenotype of WH99, with a partial activation of defenses, also suggests that tolerance is a graded trait regulated by modulating coordination completeness. This is in line with a known mode of action for redox homeostasis in drought-tolerant rice (Bhattacharjee and Dey, 2018; Samota et al., 2024).

The variety-specific antioxidant enzyme activity differences and gene expression patterns (**Figure 2**) may result from different mechanisms that each variety utilizes to adapt. A common increase in CAT activity between the two resistant varieties, 303B and HY796, is possibly an important adaptive response shared by these two varieties to break down hydrogen peroxide, essential to maintain the membrane integrity under drought (Xu et al., 2023; Su et al., 2024). The variety-specific induction of POD (only in 303B) and SOD (only in HY796), however, is a reflection of their different metabolism priorities. As an example, the high level of POD activity observed for 303B correlates to a higher abundance of genes involved in phenylpropanoid pathway and plant cell wall reinforcement (GO/InterPro) where the role of POD in the process of lignin formation becomes evident, and the specific increase observed in SOD activity by HY796 suggests that it uses glutathione-based pathways to maintain the cellular redox state, as SOD represents a first line of defense against superoxide radicals and works in synergy with the glutathione pathway (Gill and Tuteja, 2010; Jain et al., 2018; Jomova et al., 2024). Differential regulation at the transcriptomic level of *OsDSM1*/*OsCPK9*/*OsSNAC1* could be a consequence of the differential regulation of their upstream genes (e.g., transcription factors) and robustness of PPI networks. Tolerant varieties contain good hubs that are highly connected (peroxidases for 303B and SAM1 in HY796), which guarantee coordinated signal transduction and therefore synchronized gene expression. In susceptible varieties (e.g., 302B), the network is fragmented such that it does not have a coherent response to stresses.

Our proteomic results showed an obvious genotypic difference in the size of proteome remodeling under drought, where the tolerant cultivar HY796 had the most DEPs, mostly upregulated as compared with 303B and sensitive varieties (**Figure 3**). This observation can be explained by their divergent molecular strategies for drought adaptation. The proteome of HY796 appears to support this “mass mobilization” strategy with a widespread transcriptional/metabolic reprogramming indicated by the broad upregulation of proteins in pathways such as glutathione metabolism and carbohydrate metabolism, associated with a strong downregulation of photosynthetic constituents (**Figures 4**, **5**). This approach is probably dependent on the co-expression of a high number of proteins in order to rewire primary and secondary metabolism, thus maintaining redox and energy homeostasis on a whole-body level (Barros Junior et al., 2021; Urban et al., 2021). The tolerant cultivar 303B seems to adopt a more “surgical-precision” approach: it regulates a smaller number of proteins, especially those involved with intracellular transport, signaling, and some defense responses (e.g., Armadillo-domain proteins) (**Figures 4**, **5**). The robustness of 303B’s PPI network, despite having fewer DEPs, with high-connectivity hub proteins (**Figure 7**), indicates that an efficient, targeted adjustment of key regulatory nodes can be equally effective. In contrast, the sensitive variety 302B showed a disorganized DEP profile, reflecting a failure to coordinate an effective response, while WH99’s intermediate profile indicates a partial activation of defense mechanisms (**Figures 4**, **5**). Therefore, the quantity of DEPs is not a direct indicator of tolerance strength but rather a reflection of the genotype-specific tactical approach to stress management.

The InterPro enrichment analysis also provided insights into the genotype-specific molecular mechanisms of drought tolerance (**Figure 6**) (Pasandideh Arjmand et al., 2023; Aljeddani et al., 2024). The specific family of proteins or functional domain enriched for every variety reflects the genetic background and priority given to respond to stresses. The upregulation of Armadillo-type fold domains (involved in intracellular transport) and the downregulation of cytochrome P450 domains in the tolerant cultivar 303B suggest that this variety is implementing a more precise approach to protein traffic and efficient signal transmission, rather than a broad metabolic overhaul. This is similar to 303B’s robust PPI network hub proteins (e.g., peroxidases) that coordinate targeted responses. In contrast, the HY796 enrichment for carbohydrate hydrolase and redox cofactor binding domains points to a mass mobilization strategy, favoring energy metabolism and detoxification, consistent with the high expression of glutathione pathways and SOD activity (Cassier-Chauvat et al., 2023). Conversely, the sensitive variety 302B showed upregulation of lipoxygenase and phenylpropanoid-related domains, suggesting an over-activation of stress signaling and lipid peroxidation pathways that may lead to oxidative damage, rather than effective homeostasis. The moderately tolerant WH99 exhibited partial defense domain upregulation (e.g., pathogenesis-related proteins) but downregulation of heat shock domains, suggesting a loss in protein-folding ability. These InterPro patterns support the GO/KEGG results and PPI network complexity, all showing how drought resistance emerges through domain-scale reconfiguration, adapted for the respective genotypes’ metabolic advantages and disadvantages.

In addition, important binding hubs or linker proteins, which are responsible for orchestrating the drought response in different varieties, were identified from the PPI networks (**Figure 7**). In tolerant variety 303B, high degree hubs such as Os1_08946, a peroxidase, and others, are all chaperones acting as important links between the antioxidant defenses and the trafficking and signaling pathways (Liebthal et al., 2018). It is likely that these hubs are central coordinators for the fast spreading of signals across the different modules (ROS scavenging and carbohydrate metabolism). Likewise, in HY796, where the hub protein SAM1 (*S*-adenosylmethionine synthetase) links together polyamine synthesis with redox balance and energetic partitioning, suggesting its role during the maintenance of energy homeostasis in times of stress (Li et al., 2025). However, sensitive variety 302B did not have well-connected hubs with fragmented networks and a disunited response. The presence of such highly connected proteins in the tolerant varieties suggests that drought tolerance is not just about a single protein being upregulated but also the topological robustness of the interaction network, where hubs are the functional linkers for global coordination across the whole system. This is consistent with the GO/KEGG results that showed enrichment pathway related to stress signaling and metabolism and suggests that the hub protein should be targeted in subsequent breeding efforts toward improved network robustness.

## 5 Conclusion

In conclusion, our integrated analysis reveals that drought tolerance in rice is achieved through diverse molecular strategies rather than a single universal mechanism. The contrasting proteomic profiles of the two tolerant cultivars—HY796 with its extensive “mass mobilization” of metabolic pathways and 303B with its “targeted-precision” regulation of key cellular processes—demonstrate that high drought resilience can be attained through different means. Both strategies effectively coordinate physiological, transcriptional, and proteomic responses to maintain growth under stress. Therefore, the number of DEPs is not an indicator of superiority but a reflection of genetic diversity in adaptation tactics. This insight is crucial for breeding, suggesting that future efforts should aim to pyramid favorable elements from both strategic types, such as robust metabolic capacity from HY796 and efficient signaling network hubs from 303B, to develop high-yielding, drought-tolerant rice varieties.

## Data Availability

All plant materials were provided by the Rice Research Laboratory of the Chongqing Three Gorges Academy of Agricultural Sciences and are available from the corresponding authors upon reasonable request for non-commercial research purposes.The mass spectrometry proteomics data presented in the study are deposited in the ProteomeXchange repository, accession number PXD072923.
