# Supplementary material for: Integrative comparative proteomics identifies core differentially expressed proteins and pathways for drought tolerance by exploiting contrasting rice varieties
Source: Front Plant Sci. 2026 Mar 6;17:1742602. doi: 10.3389/fpls.2026.1742602 (PMC13002813; doi:10.3389/fpls.2026.1742602)
Supplement: Supplementary file 1 [file Table1.doc]

**Integrative Comparative Proteomics Identifies Core Differentially Expressed Proteins and Pathways for Drought Tolerance by Exploiting Contrasting Rice Varieties**

**The lists of supplementary materials:**

**Table S1: Primers for qRT-PCR analysis**

**Table S2: Identification of Differentially Expressed Proteins (DEPs)**

**Table S3: Gene Ontology (GO) Enrichment Analysis**

**Table S4: Kyoto Encyclopedia of Genes and Genomes (KEGG) Metabolic Pathway Analysis**

**Table S5: InterPro Enrichment Analysis**

**Table S6: Differential Protein-Protein Interaction (PPI) Network Analysis**

**Table S1 Primers for qRT-PCR analysis**

| Genes | Primer Sequence |
| --- | --- |
| *OsDSM1*-F | CAAAATGCTGGCAGACGGAC |
| *OsDSM1*-R | TTGTACTCTCTGCCTTGGCG |
| *OsCPK9*-F | AAGTCGACACCGACAAGGATG |
| *OsCPK9*-R | AACCGCTCCCTCGAGTATTG |
| *OsLG3*-F | GAGCTTCGACGACTTCCCAA |
| *OsLG3*-R | GACGACGTGATTAACAATTGAGT |
| *OsERF71*-F | CGGCTTCGCTAAAGGTGGA |
| *OsERF71*-R | ATCTCTGATTTCCGCAGCCC |
| *OsbZIP23*-F | CGCCAGAGGAAACAGGCATA |
| *OsbZIP23*-R | GGTCCAACTTGTCGGCTCAT |
| *OsSNAC1*-F | GCGAGGGGTCAAGACTGATT |
| *OsSNAC1*-R | GCACCCAATCATCCAACCTGA |
| *OsMYB48-1*-F | ATAATGAGAAAGGGCCCGTGG |
| *OsMYB48-1*-R | GCTGCGCTGCAAACCTGAC |
| *OsWRKY55*-F | AACAATGTCTCCTGTGCCGA |
| *OsWRKY55*-R | GCGCTTCTCTTTCCTTGAGC |
| *OsbZIP62*-F | CTCAGGGAGCACAAGGCTGTT |
| *OsbZIP62*-R | TGGTTCCAGCTTCTGCGTCA |
| *OsActin*-F | CCTGGCAGTATGAAGGTAGTTG |
| *OsActin*-R | GAAGCACTTCATGTGGACGAT |
